# Rational design of a hypoallergenic Phl p 7 variant for immunotherapy of polcalcin-sensitized patients

**DOI:** 10.1038/s41598-019-44208-0

**Published:** 2019-05-24

**Authors:** Marianne Raith, Doris Zach, Linda Sonnleitner, Konrad Woroszylo, Margarete Focke-Tejkl, Herbert Wank, Thorsten Graf, Annette Kuehn, Mariona Pascal, Rosa Maria Muñoz-Cano, Judith Wortmann, Philipp Aschauer, Walter Keller, Simone Braeuer, Walter Goessler, Ines Swoboda

**Affiliations:** 1 Biotechnology Section, FH Campus Wien, University of Applied Sciences, Campus Vienna Biocenter, Vienna, Austria; 2 Department of Biomedical Analytics, University of Applied Sciences Wiener Neustadt, Wiener Neustadt, Austria; 3 Division of Immunopathology, Department of Pathophysiology and Allergy Research, Center for Pathophysiology, Infectiology and Immunology, Medical University of Vienna, Vienna, Austria; 4 Department of Infection and Immunity, Luxembourg Institute of Health, Esch-sûr-Alzette, Luxembourg; 5 Hospital Clínic de Barcelona, Immunology Department, CDB, IDIBAPS, University of Barcelona, Barcelona, Spain; 6 Hospital Clínic de Barcelona, Allergy Unit, Pneumology Department, ICR, IDIBAPS, University of Barcelona, Barcelona, Spain; 7 Institute of Molecular Biosciences, BioTechMed, University of Graz, Graz, Austria; 8 Institute of Chemistry, Analytical Chemistry, University of Graz, Graz, Austria

**Keywords:** Molecular medicine, Immunology

## Abstract

Polcalcins are important respiratory panallergens, whose IgE-binding capacity depends on the presence of calcium. Since specific immunotherapy is not yet available for the treatment of polcalcin-sensitized patients, we aimed to develop a molecule for efficient and safe immunotherapy. We generated a hypoallergenic variant of the grass pollen polcalcin Phl p 7 by introducing specific point mutations into the allergen’s calcium-binding regions. We thereby followed a mutation strategy that had previously resulted in a hypoallergenic mutant of a calcium-binding food allergen, the major fish allergen parvalbumin. Dot blot assays performed with sera from Phl p 7-sensitized patients showed a drastically reduced IgE reactivity of the Phl p 7 mutant in comparison to wildtype Phl p 7, and basophil activation assays indicated a significantly reduced allergenic activity. Rabbit IgG directed against mutant rPhl p 7 blocked patients’ IgE binding to wildtype Phl p 7, indicating the mutant’s potential applicability for immunotherapy. Mass spectrometry and circular dichroism experiments showed that the mutant had lost the calcium-binding capacity, but still represented a folded protein. *In silico* analyses revealed that the hypoallergenicity might be due to fewer negative charges on the molecule’s surface and an increased molecular flexibility. We thus generated a hypoallergenic Phl p 7 variant that could be used for immunotherapy of polcalcin-sensitized individuals.

## Introduction

Allergic reactions caused by pollen are a major burden for sensitized individuals. Avoidance of the allergen source is impossible during the flowering period of the plants while symptomatic treatment can only reduce symptoms. The only disease-modifying treatment is allergen-specific immunotherapy. Specific immunotherapy is based on the repeated administration of increasing doses of an extract of the causative pollen source over 3–5 years with the aim to induce an immunological tolerance resulting in a long-term relief of symptoms. Immunotherapy has been shown to be highly effective in patients with seasonal pollinosis and seasonal asthma^1–3^. However, it bears the risk of undesired side effects, leading often to therapy discontinuation. Thus, there is a medical need for advanced and safe immunotherapy. One strategy to lower the risk of side effects is the genetic engineering of recombinant hypoallergenic allergen derivatives with reduced IgE-binding capacity, but retained T cell reactivity^4^. However, for this the knowledge of the amino acids involved in the formation of the IgE-binding epitopes is crucial. Important allergens belong to the family of calcium-binding proteins, among them ubiquitous pollen allergens (present in pollen of trees, grasses, and weeds) or important food allergens (e.g., from fish)^5^. These proteins have two or more calcium-binding domains (EF-hand motifs), which always consist of a 12 amino acid long loop flanked by two α-helices^6^. It has been demonstrated that the calcium-depleted forms of these proteins, the so called apoforms, display significantly reduced IgE reactivity as compared to the calcium-bound forms (holoforms), indicating that the presence of calcium effects the allergens’ IgE-binding capacity^7,8^. Previously, it has been shown for the major fish allergen parvalbumin that mutation of the first and third amino acids of the two calcium-binding domains, which are known to be involved in calcium-coordination, can successfully lead to hypoallergenic molecules^9,10^. The therapeutic potential of hypoallergenic carp parvalbumin for treatment of fish allergy has recently been shown in a European Union–funded project (FAST)^11^.

We hypothesized that the same mutation strategy that resulted previously in a hypoallergenic food allergen can also be applied to calcium-binding respiratory allergens. The aim of the present study was to provide the proof of principle for this strategy by modification of a respiratory allergen, the calcium-binding pollen allergen Phl p 7. The timothy grass pollen allergen Phl p 7 belongs to the polcalcins, a family of two EF-hand, highly cross-reactive allergens present in pollen of all flowering plants^8,12^. We selected Phl p 7 as a model allergen, because it contains more IgE-binding epitopes than the other members of the polcalcin family^12,13^. Exchange of the polar first and third amino acids of the two calcium-binding sites of Phl p 7 for non-polar alanines indeed resulted in a hypoallergenic molecule with reduced IgE-binding activity and reduced basophil-activating capacity. We further showed that the reduced allergenic activity of the Phl p 7 mutant is associated with a loss of calcium-binding capacity, but that the molecule’s fold is not affected. Our data indicate that amino acids in the same positions of the calcium-binding regions as in parvalbumins are also involved in the formation of IgE-binding epitopes in case of polcalcins and that mutation of these amino acids leads to the generation of hypoallergenic polcalcins, which could be used as novel therapeutic agents for immunotherapy of polcalcin-sensitized patients.

## Results

### Generation of recombinant Phl p 7 mutant

Analogous to the previous successful development of hypoallergenic fish parvalbumins^9,10^, four calcium-coordinating amino acids in the highly conserved calcium-binding domains of Phl p 7, namely aspartic acid (D) at position 1 and asparagine (N) at position 3 of the first domain as well as aspartic acids at positions 1 and 3 of the second domain were replaced by alanines (A) (Fig. S1). Wildtype and mutant Phl p 7 were expressed in *Escherichia coli* and purified to homogeneity (Fig. S2A). MALDI-TOF mass spectrometry analysis revealed molecular masses of 8.53 kDa for the Phl p 7 wildtype protein and of 8.36 kDa for the Phl p 7 mutant protein (Fig. S2B), which corresponded to the theoretical molecular masses of 8.54 kDa and 8.37 kDa calculated based on the amino acid sequences.

### The mutant variant of rPhl p 7 lacks IgE reactivity and shows reduced allergenic activity *in vitro*

**Figure 1:**
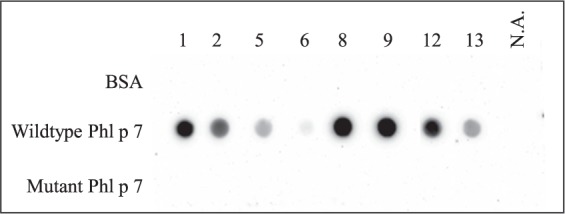
Mutant rPhl p 7 shows reduced IgE reactivity. Wildtype and mutant rPhl p 7 and, for control purposes, BSA were dotted on a nitrocellulose membrane. The membrane was cut into strips which were exposed to individual sera from Phl p 7 allergic patients (1, 2, 5, 6, 8, 9, 12, 13) and from a patient sensitized to an unrelated allergen source (N.A.).

**Figure 2:**
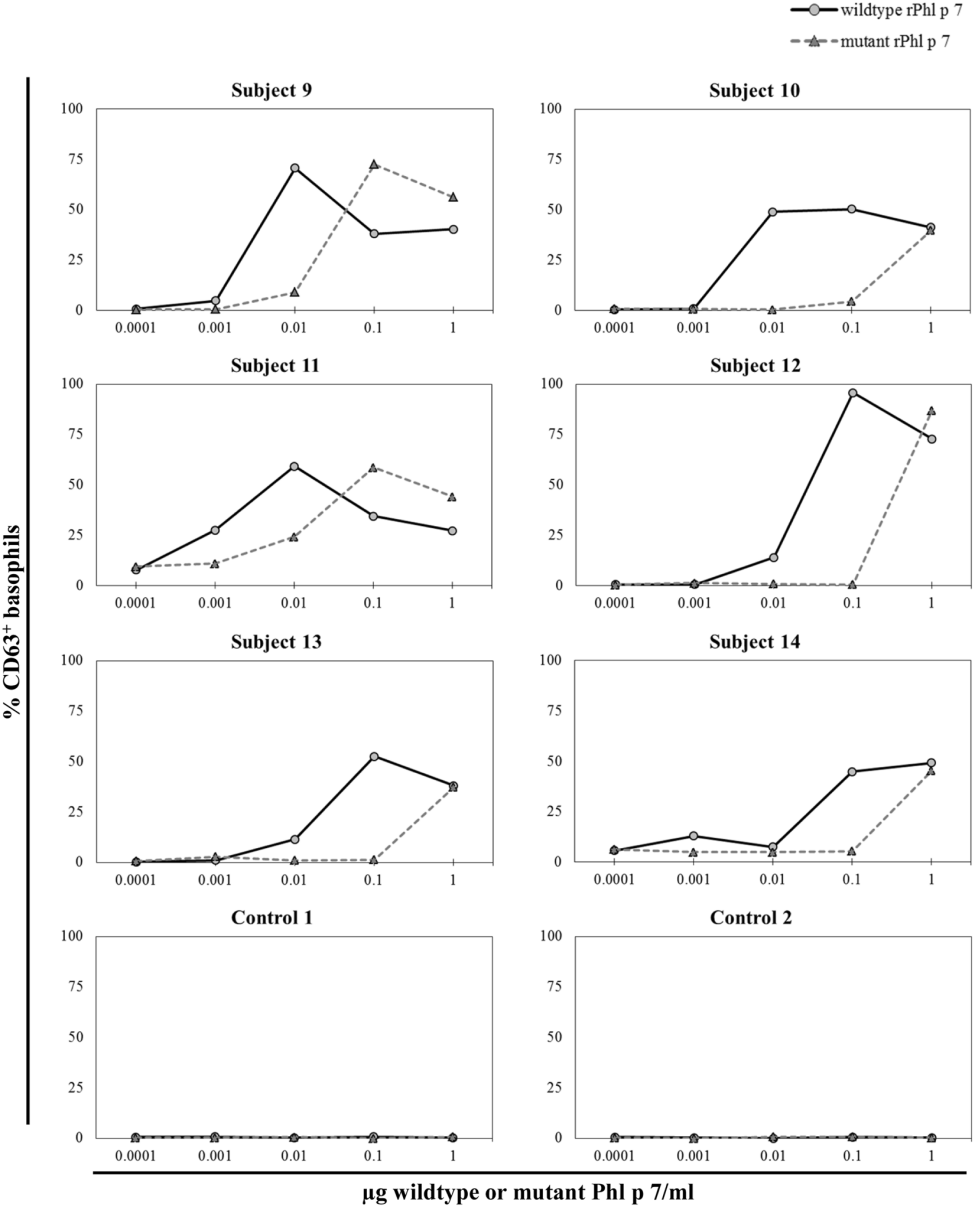
Mutant rPhl p 7 has a reduced capacity to activate patients‘ basophils. Basophil activation was determined by measuring the upregulation of CD63 by flow cytometry after incubation of whole blood from six Phl p 7-sensitized patients and two non-atopic control individuals with increasing concentrations of wildtype and mutant rPhl p 7 from 1 × 10^−4^ to 1 µg/ml (x-axis). The percentage of CD63 positive basophils is displayed (%CD63+; y-axis).

For analysis of the molecules’ IgE reactivity, dot blot assays were performed with recombinant wildtype and mutant Phl p 7 proteins using sera of eight Phl p 7-sensitized patients, which displayed Phl p 7-specific IgE levels above 4.5 kU_A_/L (as measured by ImmunoCAP, Table 1). All patients’ sera showed strong IgE binding to the wildtype protein, whereas none of the sera displayed IgE reactivity to the mutant protein (Fig. 1, a less exposed dot blot is shown in Fig. S3). In order to evaluate the mutant’s allergenic activity, basophil activation tests were performed by assessing the capacity of the wildtype and mutant proteins to induce CD63 expression on basophils. Wildtype Phl p 7 induced strong and dose-dependent upregulation in CD63 expression on basophils from six Phl p 7-sensitized patients (Fig. 2). This basophil activation was patient-dependent and did not correlate with Phl p 7-specific IgE levels determined by ImmunoCAP (Table 1). However, mutant rPhl p 7 displayed an at least 10-fold reduced allergenic activity in all tested patients. Also, this reduction of basophil activation was patient-dependent: whereas in patients 10, 12, 13 and 14 only rPhl p 7 mutant concentrations of 1 µg/ml could activate the basophils, mutant concentrations of 0.1 µg/ml led to basophil activation in patients 9 and 11. No basophil activation was induced by wildtype or mutant Phl p 7 in six individuals sensitized to unrelated allergen sources (food) serving as controls (two representative experiments are shown in Fig. 2).

**Table 1:** Demographic and clinical characterization of Phl p 7-sensitized patients.

Subject	Age/Gender	Symptoms	other respiratory allergen sources	Total IgE (kU/L)	Phl p 7 (kU_A_/L)
1	13/M	rhinitis, asthma	cypress, plane tree, *Parietaria spp*.	642	48.3
2	52/F	rhinitis	cypress, olive	132	47.8
3	8/F	rhinitis	cypress, plane tree, house dust mite, epithelia, *Alternaria spp*.	3,102	0.53
4	13/M	rhinitis	cypress, plane tree, olive, epithelia	1,107	0.57
5	11/M	rhinitis	epithelia	730	26.7
6	13/M	asthma	cypress, olive, *Parietaria spp*., house dust mite, epithelia, *Alternaria spp*.	495	9.87
7	34/F	rhinitis	plane tree	750	2.12
8	17/F	rhino-conjunctivitis	plane tree, olive, *Parietaria* *spp*., chenopodium, mugwort, house dust mite, epithelia	1,093	>100
9	10/F	rhinitis, asthma	plane tree, olive, elm, *Parietaria spp*., mugwort	559	>100
10	42/M	rhinitis	not known	403	4.12
11	23/F	rhino-conjunctivitis	plane tree, birch, olive, chenopodium, *Parietaria spp*., mugwort, house dust mite, *Blatella spp*.	92.6	2.59
12	33/F	rhinitis	cypress, plane tree, birch, olive, *Parietaria spp*., chenopodium, mugwort, plantago, house dust mite, *Blatella spp*., epithelia	210	12.9
13	45/M	rhinitis, asthma	not known	63.6	14.7
14	32/M	rhinitis	plane tree, birch, *Parietaria spp*., chenopodium, mugwort, plantago, house dust mite, *Alternaria spp*.	203	4.5

### IgG antibodies to the rPhl p 7 mutant protein block patients’ IgE binding to wildtype Phl p 7

Immunization of New Zealand White rabbits showed that mutant rPhl p 7 induced specific IgG antibodies that recognized not only the mutant, but also the Phl p 7 wildtype protein in ELISA (Fig. S4). To further test if these IgG antibodies have the potential to inhibit binding of patients’ Phl p 7-specific IgE antibodies, we performed ELISA competition experiments using sera from eight Phl p 7-sensitized patients with high titers of Phl p 7-specific IgEs (Table 1) and from an individual sensitized to an unrelated allergen source. Anti-mutant rabbit antiserum (at 1:100 dilutions) caused a strong reduction of IgE binding to ELISA coated rPhl p 7 wildtype protein, ranging between 58.5% and 86.5% (Table 2). This finding indicates that mutant-specific IgG antibodies do have the potential to block the binding of patients’ IgE antibodies to the rPhl p 7 wildtype allergen, a prerequisite for a molecule potentially used in immunotherapy^14^.

**Table 2:** Rabbit anti-rPhl p 7 mutant antibodies inhibit patients’ IgE binding to the Phl p 7 wildtype protein.

Patient	rabbit pre-immune serum (OD 405 nm)	rabbit anti-rPhl p 7 mutant serum (OD 405 nm)	% of inhibition
1	1.677 ± 0.012	0.227 ± 0.001	86.5
2	1.130 ± 0.026	0.255 ± 0.001	77.4
5	2.270 ± 0.012	0.943 ± 0.057	58.5
6	0.851 ± 0.028	0.198 ± 0.005	76.7
8	1.470 ± 0.027	0.327 ± 0.007	77.8
9	1.375 ± 0.012	0.220 ± 0.003	84.0
12	0.740 ± 0.009	0.197 ± 0.020	73.4
13	0.458 ± 0.017	0.141 ± 0.000	69.3
N.A.	0.115 ± 0.004	0.158 ± 0.004	0

ELISA plate-bound rPhl p 7 wildtype was preincubated with 1:100 diluted rabbit anti-rPhl p 7 mutant serum or with the preimmune serum and subsequently exposed to the sera from eight Phl p 7-sensitized patients or from a patient sensitized to an unrelated allergen source (N.A.). IgE binding was measured by ELISA and mean OD values at 405 nm and percentage inhibition of IgE binding is displayed.

### The calcium binding capacity of mutant rPhl p 7 is abolished while the overall secondary structure is retained

**Figure 3:**
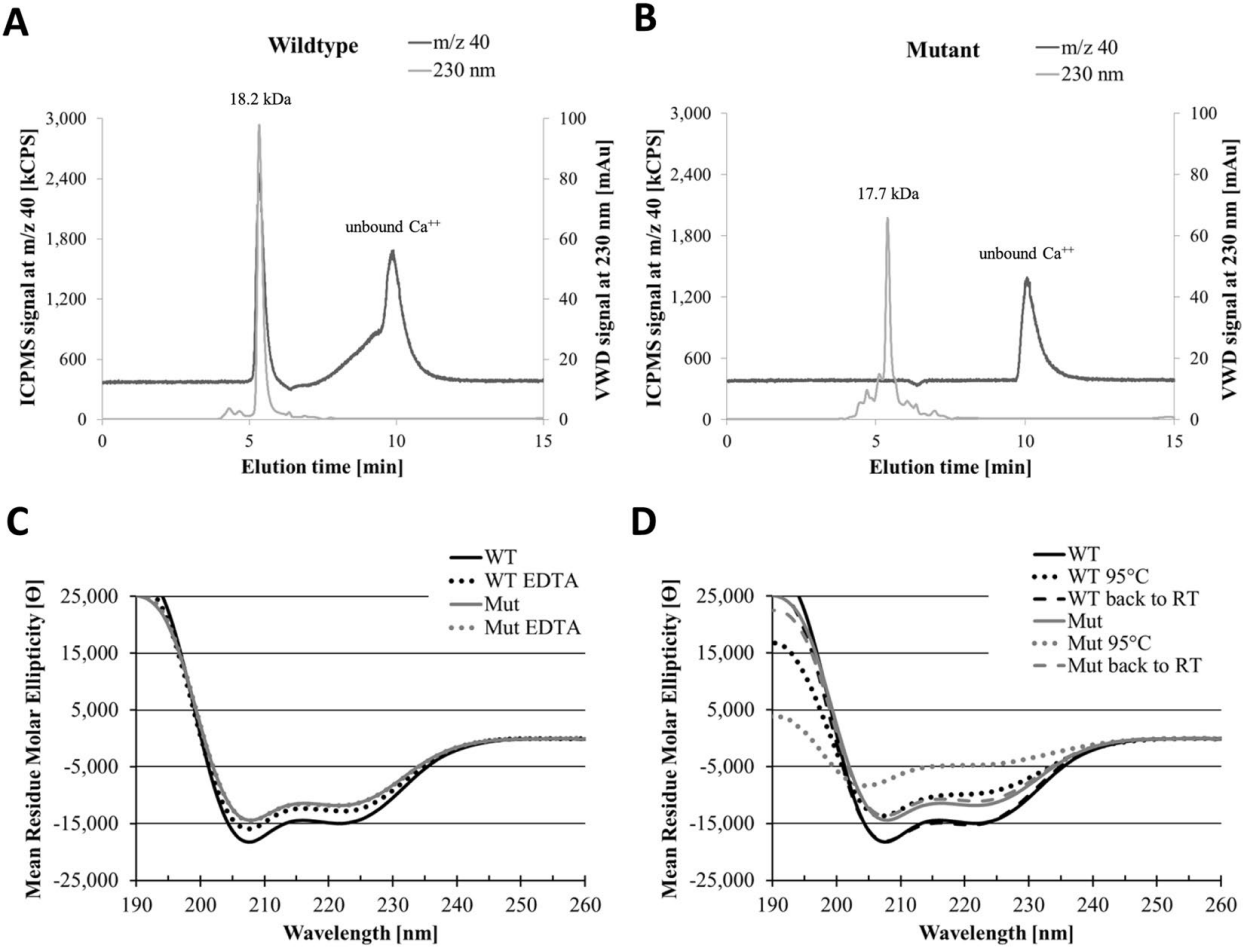
Mutant rPhl p 7 lacks calcium-binding capacity, but represents a folded protein with slightly reduced thermal stability. Size exclusion chromatography coupled to ICPMS was performed with wildtype (**A**) and mutant (**B**) rPhl p 7. UV chromatograms (VWD signal) at 230 nm (grey lines) are given in milli arbitrary units (mAu, right y-axis) and the corresponding calcium ion count mass spectrometry traces (m/z 40, black lines, left y-axis) are shown in kilocounts per second (kCPS). The x-axis represents the elution time in minutes (min). (**C**) CD spectra of wildtype (WT, black) and mutant (Mut, grey) rPhl p 7 recorded under native conditions (continuous lines) or after addition of EDTA (dotted lines). Note that the rPhl p 7 Mut ETDA line is fully congruent with the rPhl p 7 Mut line. (**D**) Temperature ramping experiments from room temperature (continuous lines) to 95 °C (dotted lines) and back to room temperature (dashed lines). The spectra are expressed as the mean residue ellipticity (deg*cm^2^*dmol^−1^; y-axis) at a given wavelength (x-axis).

To analyze whether the reduced allergenic activity of the rPhl p 7 mutant protein is associated with a loss of calcium-binding capacity, size exclusion chromatography experiments coupled to an UV-detector and an ICPMS detector were performed with the rPhl p 7 wildtype and mutant proteins. Both proteins eluted as dimers with apparent molecular masses of 18.2 kDa (wildtype; elution time at 5.33 minutes) and 17.7 kDa (mutant; elution time at 5.40 minutes) (grey lines in Fig. 3A,B). However, whereas the wildtype protein co-eluted with a strong calcium peak (Fig. 3A, black line), no calcium co-eluted with the mutant protein, indicating that the mutant had lost the calcium-binding ability (Fig. 3B, black line). In case of both proteins unbound calcium eluted at an elution time of 9.89 and 10.07 minutes (Fig. 3A,B).

As a next step circular dichroism (CD) analyses were performed with the rPhl p 7 wildtype and mutant proteins to investigate whether the loss of calcium-binding capacity of the mutant resulted in an altered secondary structure. With minima at 208 and 222 nm and a maximum below 200 nm, the wildtype protein displayed a spectrum typical for folded α-helical proteins. The spectrum of the mutant showed less pronounced minima at 208 and 222 nm, indicating that the mutant also displayed a folded protein with a slightly lower α-helical content (Fig. 3C, continuous lines). Depletion of calcium by the addition of the calcium-chelator EDTA resulted in a spectrum with less pronounced minima in case of the wildtype protein (Fig. 3C dotted lines), indicating that the folding is influenced by the depletion of calcium. As expected, owing to the lack of calcium-binding capability, the mutant protein’s spectrum did not change after addition of EDTA (Fig. 3C, dotted lines).

In order to compare the proteins’ stability, we performed temperature scan experiments where the wildtype and mutant proteins were heated up to 95 °C and re-cooled to 20 °C. At 95 °C, the spectrum of the wildtype protein still showed minima at 208 and 222 nm (Fig. 3D, black dotted line), indicating a folded protein. In contrast, the spectrum of the mutant protein had changed: one minimum had shifted toward 205 nm and the second one was hardly visible anymore, indicating a partial denaturation of the protein (Fig. 3D, grey dotted line). Determination of the melting temperatures showed that the wildtype protein’s melting temperature (89.2 °C ± 0.5 °C) was around 10 °C higher than the melting temperature of the mutant (79.7 °C ± 1.0 °C) (data not shown). This also demonstrated the slightly lower stability of the mutant protein. However, it was interesting to see that both proteins were able to completely refold after re-cooling to 20 °C (Fig. 3D, back to RT, dashed lines). The fact that the mutant protein regained its secondary structure after re-cooling shows that the loss of calcium-binding capacity caused by the exchange of four calcium-coordinating amino acids only had a limited effect on the overall structure and on the thermal stability of the protein.

### Mutant rPhl p 7 displays a reduction in the negative charge of the surface and an increase in the molecular flexibility as compared to wildtype Phl p 7

**Figure 4:**
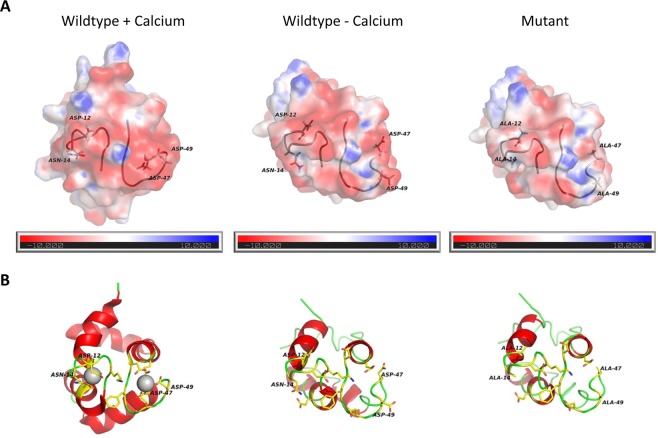
Mutant Phl p 7 displays a reduced negative surface charge. Electrostatic surface potential (**A**) and ribbon diagram (**B**) of Phl p 7 wildtype with calcium (PDB code: 2lvk), without calcium (PDB code: 2lvi) and mutant Phl p 7 (homology model based on 2lvi). Calcium-binding loops and modified amino acids are marked (Asp 12, Asn 14, Asp 47 and Asp 49). (**A**) The color scheme of the electrostatic surface potential ranges from red via white to blue for −10 to +10 kT/e. (**B**) Ribbon diagrams show helices (red), loop regions (green), calcium-interacting residues (yellow), and bound calcium (grey). All structures are aligned and in the same orientation.

Since ICPMS and CD spectroscopy results indicated that mutant rPhl p 7 is not able to bind calcium, we expected that the electrostatic potential of the mutant would resemble the electrostatic potential of the calcium-depleted wildtype molecule. To investigate the theoretical effects of the mutations on the charge distribution, a homology model of the rPhl p 7 mutant was generated based on the NMR structure of the calcium-free Phl p 7 wildtype molecule (PDB code 2lvi)^15^. Comparison of the sensitizing calcium bound form (holoform; PDB code 2lvk) and the calcium-free form (apoform; PDB code 2lvi) of wildtype Phl p 7 showed significant differences in the electrostatic potential of the wildtype molecule depending on the presence or absence of calcium (Fig. 4A). Interestingly, differences in the charge distribution could also be observed between the calcium-depleted wildtype protein and the mutant protein, which displayed a less negative charge than the apoform of wildtype Phl p 7 (Fig. 4A), indicating that the surface charge is not only influenced by the presence of calcium. As can be seen in the ribbon diagrams of the three proteins, the reduced negative surface charge of the mutant occurs predominantly in the areas of both calcium binding loops (Fig. 4B).

**Figure 5:**
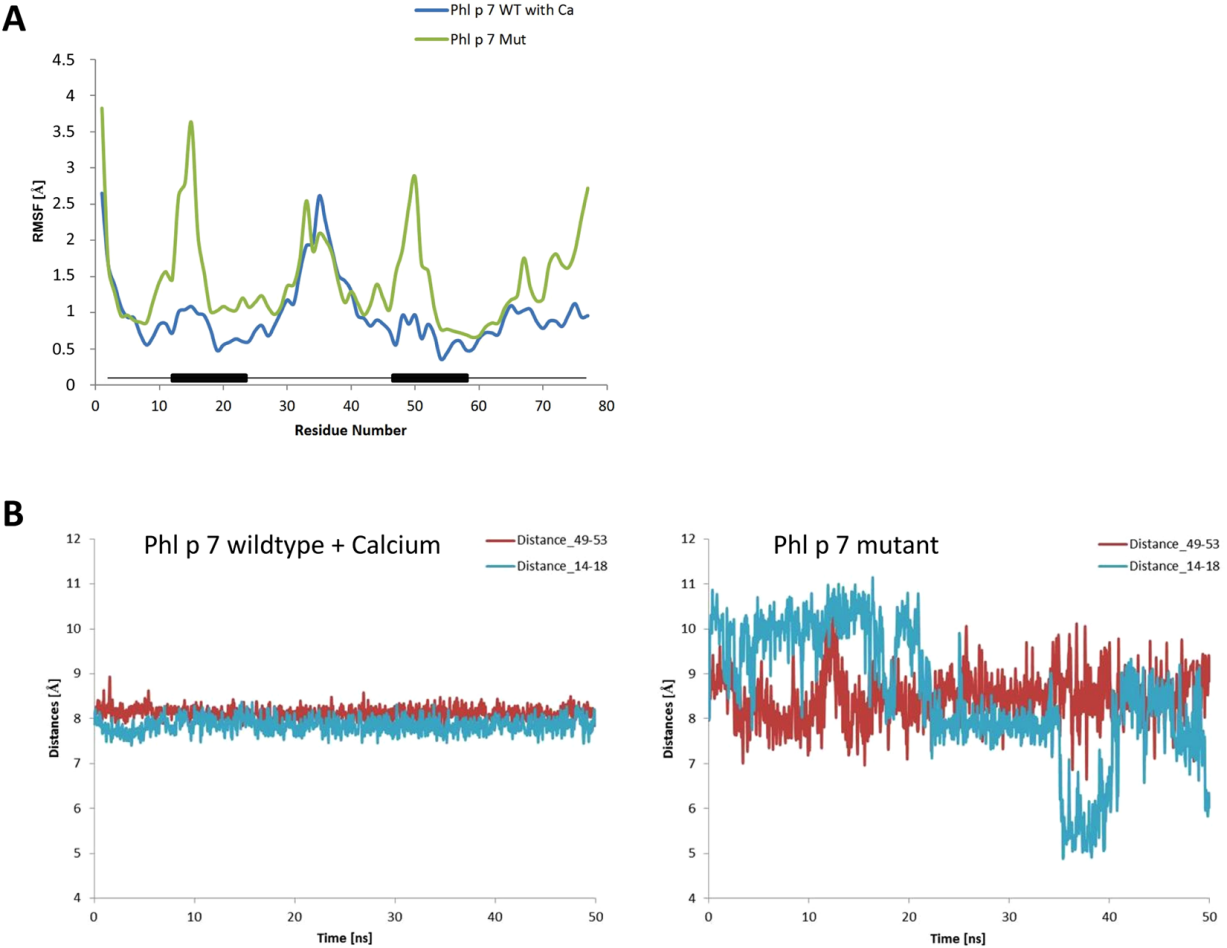
Increased structural flexibility in the calcium-binding regions of mutant Phl p 7. (A) Root mean square fluctuations (RMSF) of the protein backbone Cα atoms obtained in 50 ns MD simulations for calcium-bound wildtype Phl p 7 and for the Phl p 7 mutant. The RMSF in Ångström (Å, y-axis) are displayed for each residue (x-axis). The calcium-binding loops (residues 12–23 and 47–58) are marked with black boxes. (**B**) Distances (y-axis, in Å) between the Cα atoms of the opposite calcium-binding residues 14–18 (blue line) and 49–53 (red line) as a function of time (x-axis, in ns) of calcium-bound Phl p 7 wildtype (left panel) and of the mutant Phl p 7 (right panel).

To further analyze if the effects of the introduced mutations and the consequential calcium-depletion have an impact on the structural integrity and flexibility of Phl p 7, molecular dynamic simulations were performed. Root mean square fluctuation analysis per residue (RMSF, Fig. 5A) showed that the mutant Phl p 7 displayed larger RMSF peaks than the wildtype molecule in the calcium-binding loops (residues number 12–23 and 47–58), indicating a greater mobility of the mutant protein in these areas. Furthermore, analysis of the root mean square deviation (RSMD, Fig. 5B) between residues involved in calcium binding and positioned opposite to the Ca-binding sites, namely the third and seventh amino acids in each calcium-binding loop (residues 14–18 and 49–53), of the Phl p 7 wildtype holoform (left panel) and the Phl p 7 mutant (right panel) showed that the analyzed residues display a considerably higher flexibility in the mutant. This clearly indicates greater conformational freedom of the calcium-binding loops in the mutant as compared to wildtype Phl p 7.

## Discussion

In this study we produced a hypoallergenic variant of the EF-hand calcium-binding grass pollen allergen Phl p 7 by following the same strategy that had already previously resulted in the development of a hypoallergenic derivative of the calcium-binding fish allergen parvalbumin^11,12^. In both cases the first and third amino acids of the two calcium-binding regions, aspartic acid and/or glutamic acid, which are known to be involved in calcium-coordination, were substituted by alanines. Dot blot experiments showed that the rPhl p 7 mutant displayed a reduced IgE-binding capacity and basophil activation tests indicated also a reduced biological activity. Since there is no cross-reactivity between calcium-binding fish allergens and pollen allergens, IgE-binding epitopes of parvalbumins and polcalcins are certainly different. Our results show that mutation of four amino acids localized at the same positions in the calcium-binding regions destroys the IgE-binding capacity not only of food allergens, but also of respiratory allergens. This demonstrates the essential role of the four calcium-binding amino acids in the formation of the IgE-binding epitopes and paves the way for the generation of further hypoallergenic, calcium-binding allergens.

To understand the molecular basis for the hypoallergenicity, we investigated the calcium-binding capacity of wildtype and mutant rPhl p 7. Size exclusion chromatography coupled to ICPMS showed that calcium was bound to recombinant wildtype Phl p 7, proofing that wildtype rPhl p 7 was present as the holoform. In contrast, no calcium was bound to mutant rPhl p 7. This indicated that the introduced mutations had destroyed the molecule’s ability to bind calcium, which had then resulted in the reduced IgE-binding capacity of the mutant.

Furthermore, we compared the mutant’s structure with the structure of the wildtype protein by CD-spectroscopy and observed that the rPhl p 7 mutant protein was still folded and that the far-UV spectrum still displayed the typical characteristics of an α-helical protein. Temperature scan experiments, where the proteins were heated up to 95 °C and re-cooled to 20 °C, showed that the mutant unfolded at a temperature that was 10 °C lower than the melting temperature of wildtype Phl p 7, which indicated a partial heat-induced destabilization of the mutant. Since the mutant fully regained its α-helical fold after cooling back to 20 °C, the exchange of the four calcium-coordinating amino acids apparently only had a limited effect on the structure and thermal stability of the molecule. However, *in silico* determination of the surface charge distribution and stability analysis by means of Molecular Dynamics, indicated reduced negative surface charges and increased molecular flexibility of the mutant protein. Thus, we conclude that calcium-depletion leading to the hypoallergenicity of the mutant Phl p 7 modifies the physicochemical nature of the molecule and not its overall structure. Since the overall structure of the mutant and the wildtype molecule are very similar, IgG antibodies generated against the mutant can also recognize the wildtype protein and can prevent IgE binding to the wildtype allergen (Table 2).

Recently, Focke-Tejkl *et al*. used a different strategy, namely the concept of peptide carrier fusion vaccines, for the development of an engineered vaccine candidate for grass pollen immunotherapy^16^. This hypoallergenic vaccine consists of non-allergenic peptides of the major allergens from timothy grass pollen, Phl p 1, Phl p 2, Phl p 5 and Phl p 6 fused to the hepatitis B-derived PreS domain as a peptide carrier^16^. Since Phl p 7 represents a minor allergen for grass pollen allergic patients, peptides of Phl p 7 were not included in this timothy grass pollen vaccine. So far, immunotherapies using grass pollen extracts are not recommended for patients sensitized to Phl p 7, since this molecule is underrepresented in the therapeutic extracts resulting in limited success of immunotherapy in these cases^17^. Since polcalcins represent highly cross-reactive panallergens^18^, and Phl p 7 contains most of the IgE-binding epitopes of this group of allergens^14^, the development of a Phl p 7 vaccine is of importance.

So far two strategies were applied for the generation of a Phl p 7-based vaccine. One was a peptide approach, where either the first or the second half of Phl p 7 was coupled to KLH as carrier. However, even though these peptides had a low IgE-binding capacity, rabbit IgG antibodies generated against the peptides had no or only a very low capacity to inhibit patients’ IgE binding to Phl p 7^19^. These findings suggest that a successful Phl p 7 vaccine should not be based on peptides. Our Phl p 7 mutant showed reduced allergenic activity and induced in rabbits the generation of IgG antibodies which were able to block patients’ IgE binding to the wildtype allergen. The importance of the induction of blocking IgGs for a successful immunotherapy has been shown in several studies^20^.

The second previously tried approach was also based on the concept of hypoallergenic allergen derivatives and a mutant of Phl p 7 was developed by exchanging the 5^th^ and 12^th^ amino acids in both calcium-binding regions^19^. However, this molecule still displayed IgE-binding capacity^19^. It is known that calcium-binding in the loops of the EF-hand motifs is coordinated by highly conserved amino acids in positions 1, 3, 5, 7, 9 or 12^21^. Mutating the first and third amino acid of the EF-hand motifs apparently had a more striking effect on the destruction of the IgE-binding epitopes. Thus, it can be suggested that this mutant rPhl p 7 molecule could be a promising candidate for immunotherapy of Phl p 7 sensitized patients.

In summary, the lost calcium binding capacity of the mutant Phl p 7 leads to a reduced thermal stability, reduced surface charges and a higher flexible structure of the Phl p 7 protein, thus leading to a reduced IgE reactivity. In general, this study shows that mutagenesis of specific amino acids involved in calcium-binding resulted in a hypoallergenic Phl p 7 molecule which has the potential to be used for specific immunotherapy of patients sensitized to calcium-binding pollen allergens. Furthermore, we suggest that the same mutation strategy could also be applied to other polcalcins, and even to other calcium-binding allergens identified in house dust mite, cockroach and cattle^22–24^.

## Material and Methods

### Patient selection and determination of total and allergen-specific IgE

Sera from 14 patients (7 males, 7 females) with a positive case history of type I allergy to grass pollen and with specific IgE to Phl p 7 (Table 1) and from seven atopic patients sensitized to unrelated allergen sources were analyzed in an anonymized manner. Total and rPhl p 7-specific IgE levels were measured by ImmunoCAP (Phadia-Thermo Fisher Scientific, Uppsala, Sweden). Specific IgE levels >0.35 kU_A_/L were considered positive. All patients agreed to participate in this study and gave informed consent. The study was approved by the ethics committee of the Hospital Clínic de Barcelona (approval number 2011/6605). We confirm that all methods were performed in accordance with the relevant guidelines and regulations.

### Generation of recombinant Phl p 7 variants

A mutant of Phl p 7 was designed, where aspartic acid (D) at position 1 and asparagine (N) at position 3 of the first calcium-binding domain as well as aspartic acids at positions 1 and 3 of the second domain were replaced by alanines (A) (Fig. S1). Genes codon-optimized for expression in *Escherichia coli* coding for wildtype and mutant Phl p 7 were synthesized and cloned into the *NdeI* and *EcoRI* restriction sites of the expression vector pET17b (GenScript, Piscataway, NJ). Recombinant proteins were expressed in *E. coli* strain BL21(DE3) after addition of isopropyl-D-thiogalactoside (0.5 mM) as soluble proteins. Bacterial cell pellets were resuspended in 10 mM TrisHCl, 0.1% Triton X-100 and proteins were purified by anion-exchange chromatography using HiTrap Q FF columns (GE Healthcare) with a linear salt gradient (0–0.5 M NaCl).

### SDS-PAGE and dot blot

The purified recombinant proteins (2 µg each) were separated on 15% Tris-glycine SDS-PAGEs^25^ and stained with Coomassie brilliant blue. For the dot blot, 1 µg wildtype or mutant rPhl p 7 protein or, for control purposes, BSA was dotted onto nitrocellulose membranes. The membranes were blocked with 5% BSA in PBS-T (12.7 mM Na_2_HPO_4_, 2.2 mM KH_2_PO_4_, 140 mM NaCl, 0.5% Tween 20) for 1 hour at room temperature and incubated overnight at 4 °C with individual patient sera (diluted 1:10 in PBS-T). Membranes were then incubated with a horseradish peroxidase-labeled anti-human IgE antibody (0.5 µg/ml Southern Biotech, Birmingham, AL) in PBS-T for 1 hour at RT and bound IgE antibodies were detected using SuperSignal West Pico Chemiluminescent Substrate (Thermo Fisher Scientific) on a FluorChem E Protein simple device (Biozym Scientific GmbH, Hessisch-Oldendorf, Germany).

### Matrix-assisted laser desorption ionization-time of flight (MALDI-TOF) analysis

Wildtype and mutant rPhl p 7 proteins were analyzed in a linear mode with a microflex MALDI-TOF instrument (Bruker, Billerica, MA) using α-cyano-4 hydroxy-cinnamic acid (dissolved in 60% acetonitrile, 0.1% trifluoroacetic acid) as a matrix. For sample preparation, protein and matrix solution were mixed in equal amounts and deposited on the target. Generated spectra were mass-calibrated using rBet v 1 (Biomay, Vienna, Austria) as a standard.

### Basophil activation assay

Basophil activation tests were performed using the Flow Cast® kit (Bühlmann, Schönenbuch, Switzerland) according to manufacturer’s instructions. Whole blood from six Phl p 7-sensitized and six patients sensitized to unrelated allergen sources (food) was incubated with increasing concentrations (1 × 10^−4^ to 1 µg/ml) of the recombinant Phl p 7 molecules. Basophil activation was assessed by detecting the expression of CD63 by flow cytometry (FACS Canto II, Becton Dickinson, USA). Activated basophils were gated as SSC^low^/CCR3^+^/CD63^+^. At least 1,000 basophils were measured in all patients and the percentage of CD63-expressing basophils was calculated.

### ELISA and ELISA competition experiments

New Zealand White rabbits were immunized at Charles River Laboratories (Kisslegg, Germany) with 200 µg of the Phl p 7 mutant protein using Freund’s complete adjuvant, followed by three booster injections using Freund’s incomplete adjuvant. All experiments with rabbits were conducted at Charles River Laboratories France and performed in accordance with the Canadian Council on Animal Care (CCAC) guidelines on antibody production. All experimental protocols were approved by the French ministry (project #2016061011167092) and by the Charles River ethics committee (C2EA #41). IgG reactivity of the anti-Phl p 7 mutant antiserum to wildtype and mutant rPhl p 7 was analyzed by ELISA. For this, MaxiSorp flatbottom 96-well plates (Thermo Fisher Scientific, Waltham, MA) were coated with 0.4 µg/well wildtype or mutant rPhl p 7 in bicarbonate buffer (37 mM Na_2_CO_3_, 63 mM NaHCO_3_, pH 9.6) overnight. After washing with TBS-T (10 mM Tris-HCl with a pH 8.0, 150 mM NaCl, 0.5% Tween20), unspecific binding sites were blocked for 2.5 hours at 37 °C with 1% BSA in TBS-T. Plates were then incubated overnight with 1:2,000 diluted rabbit antisera (preimmune serum or serum taken on day 70). The binding of rabbit IgG antibodies was detected with 1:10,000 diluted AP-labeled secondary anti-rabbit antibodies (Jackson Immuno Research, West Grove, PA) and an alkaline phosphatase substrate (4-Nitrophenyl phosphatase disodium salt hexahydrate, Sigma Aldrich, St. Louis, MO). The optical density (OD) was measured at 405 nm, with a reference wavelength of 550 nm in a MultiskanTM plate reader (Thermo Fisher Scientific).

The ability of the anti-mutant antisera to block patients’ IgE binding to the recombinant Phl p 7 wildtype protein was assessed in ELISA competition assays. For this, MaxiSorp ELISA plates were coated with 0.1 µg/well of wildtype rPhl p 7 in bicarbonate buffer overnight. After washing with TBS-T and blocking with 1% BSA in TBS-T, plates were incubated overnight with a 1:100 dilution of the anti-rPhl p 7 mutant rabbit serum taken on day 70 or, for control purposes, with the corresponding preimmune serum. After incubation overnight with sera from Phl p 7 sensitized patients or from a patient sensitized to an unrelated allergen source (1:10 diluted in TBS-T) bound IgE antibodies were detected with secondary horseradish peroxidase-labeled antibodies to human IgE (KPL, Gaithersburg, MD) and a substrate solution (61 mM citric acid, 77 mM Na_2_HPO_4_, 1 mg/ml ABTS, 0.003% H_2_O_2_). Optical density (OD) was measured at 405 nm in a MultiskanTM plate reader. The percent inhibition of IgE binding was calculated as follows: % inhibition of IgE binding = 100 − (ODs/ODp) × 100, where ODs and ODp represent the extinction coefficients after preincubation with the rabbit serum and the preimmune serum, respectively. All determinations were conducted in duplicates, and results expressed as mean values.

### Inductively coupled plasma mass spectrometry (ICPMS)

For the separation and determination of calcium in wildtype and mutant rPhl p 7 proteins, an HPLC 1260 system (Agilent, Waldbronn, Germany), equipped with a variable wavelength detector (VWD) at 230 nm was used (stationary phase: Yarra SEC-X150 column (150 × 4.6 mm, 1.8 µm, Phenomenex, Aschaffenburg, Germany), mobile phase: aqueous solution of 10 mM Tris and 50 mM ammonium sulfate at pH 7.5). The flow rate was 0.3 ml/min and the temperature of the column oven 30 °C. The injection volume of the samples was 1 µl. The exit of the VWD was connected via a PEEK capillary to the nebulizer of an inductively coupled plasma mass spectrometer (ICPMS, 7700, Agilent). The ICPMS was operated in hydrogen reaction gas mode, and the Ca^2+^ signal was recorded at m/z 40. The delay time between VWD and ICPMS was 0.23 min, which was determined with a chromate solution and corrected in the chromatograms.

### Circular dichroism analysis of purified recombinant wildtype and mutant Phl p 7

Circular dichroism (CD) measurements of 0.2 µg/µl wildtype and mutant rPhl p 7 were performed in 10 mM sodium phosphate buffer at 20 °C on a Chirascan qCD spectropolarimeter (Applied Photophysics, Leatherhead, UK) using a quartz cuvette (Hellma Analytics, Müllheim, Germany) with a path-length of 1 mm. CD spectra were recorded after addition of either 0.5 mM CaCl_2_ or 5 mM EDTA (for the calcium depletion assay) from 190 to 260 nm with a resolution of 1 nm and results are the average of three repeats. Temperature scan experiments were performed according to a step-scan procedure, where the sample was heated from 20 to 95 °C (heat rate of 0.82 °C/min, tolerance of 0.2 °C). For this, the temperatures inside the cuvette were determined with a temperature sensor. Every 1 °C, single continuous wavelength spectra and after cooling back of the sample to 20 °C, three continuous wavelength spectra were recorded with the specified parameters and results are represented as the average of the three scans. The spectra were finally corrected by subtracting the buffer baseline obtained under identical conditions and smoothed using the Savitzky-Golay-Filter (window size 5). Melting temperatures were calculated with the Global 3 software (Applied Photophysics).

### Simulation modeling and calculation of molecular dynamics

A homology model of the Phl p 7 mutant was generated with the Phyre² web server^26^ based on the structure of calcium-depleted Phl p 7 (PDB Code 2lvi). This model of the Phl p 7 mutant was compared with the NMR structures of Phl p 7 wildtype with (PDB Code 2lvk) and without calcium (PDB Code 2lvi). The electrostatic potentials of the three structures were calculated with the APBS tools implemented in the UCSF Chimera package^27,28^. The projection of the electrostatic surfaces and the ribbon models were performed with PyMOL (PyMOL Molecular Graphics System, Version 2.0 Schrödinger, LLC).

Molecular Dynamics (MD) simulations were calculated with “Desmond” employing the OPLS3 force field for 50 ns with 50 ps steps at 300 K. All programs for MD simulations were used via the Maestro Environment (Maestro, Schrödinger, LLC, New York, NY, 2018). Structure preparation (including energy minimization) for MD simulation was done using the Protein Preparation Wizard. For minimization of the force field the pH was set to 7.0 ± 0.2 and the simulation was carried out in an orthorhombic box filled with explicit water. As a solvent model SPC was used. The preparation including the addition of water and Na^+^ ions for charge equilibration were performed with the “System Builder” panel. For control purposes all the simulations were repeated two times (data not shown).

## Supplementary information

Supplementary Information
