# Supplementary material for: Rational design of a hypoallergenic Phl p 7 variant for immunotherapy of polcalcin-sensitized patients
Source: Sci Rep. 2019 May 24;9:7802. doi: 10.1038/s41598-019-44208-0 (PMC6534608; doi:10.1038/s41598-019-44208-0)
Supplement: Supplementary file 1 — Supplementary Information [file 41598_2019_44208_MOESM1_ESM.pdf]

Rational design of a hypoallergenic Phl p 7 variant for immunotherapy of polcalcin-sensitized patients

Marianne Raith[1] PhD, Doris Zach[1] MSc, Linda Sonnleitner[2] BSc, Konrad Woroszylo[1] BSc, Margarete Focke-Tejkl[3] PhD, Herbert Wank[1] PhD, Thorsten Graf[4] BSc, Annette Kuehn[4] PhD, Mariona Pascal[5] PhD, Rosa Maria Muñoz-Cano[6] MD, Judith Wortmann[7] MSc, Philipp Aschauer[7] PhD, Walter Keller[7] PhD, Simone Braeuer[8] MSc, Walter Goessler[8] PhD and Ines Swoboda[1] PhD*

Supplementary figure legends

FIGURE S1 Sequence alignments of the wildtype (WT) and mutant (Mut) variants of Phl p 7 and Cyp c 1.

Aspartic acid (D) in position 1 and asparagine (N) in position 3 of the first calcium-binding domain as well as aspartic acids (D) in position 1 and 3 of the second calcium-binding domain were exchanged to alanines and are marked in red in the sequence alignment of wildtype (Phl_p_7_WT) and mutant (Phl_p_7_Mut) Phl p 7. For comparison, the previously produced Cyp c 1 wildtype and mutant variants (Cyp_c_1_WT and Cyp_c_1_Mut) are shown. Asterisk (*) indicates identical amino acids. Calcium binding domains are labeled with boxes.

FIGURE S2 Characterization of rPhl p 7 wildtype and mutant proteins.

(A) Coomassie-stained SDS-PAGE of 2 μg wildtype (WT) and mutant (Mut) proteins that had been purified under native conditions by anion-exchange chromatography using HiTrap Q FF columns (GE Healthcare) with a linear salt gradient (0-0.5 M NaCl). Molecular weights (in kDa) are indicated on the left margins. (B) Molecular mass of wildtype rPhl p 7 (WT) and mutant rPhl p 7 (Mut) determined by MALDI-TOF. The x-axis shows the mass/charge (m/z) ratio, and the signal intensity is displayed on the y-axis.

FIGURE S3 Mutant rPhl p 7 shows reduced IgE-reactivity.

Dot blot shown in Figure 1 in a lower exposure. Wildtype and mutant rPhl p 7 and, for control purposes, BSA were dotted on a nitrocellulose membrane. The membrane was cut into strips which were exposed to individual sera from Phl p 7 allergic patients (1, 2, 5, 6, 8, 9, 12, 13) and from a patient sensitized to an unrelated allergen source (N.A.).

Figure S4 Mutant rPhl p 7 induces Phl p 7 specific IgG antibodies in rabbits.

Analysis of specific IgGs to wildtype and mutant Phl p 7 in rabbit sera before (preimmune serum, dark grey bars) and after 70 days of immunization (mutant serum, light grey bars) with mutant Phl p 7. ELISA plates were coated with Phl p 7 wildtype (wildtype coating) or Phl p 7 mutant (mutant coating) proteins. Error bars represented the SD of the mean.

Figure S1

```
Phl_p_7_WT     ADDMERIFKRFDTNGDGKISLSELTDALRTLGSTSADEVQRMMAEIDTDGDGFIDFNEFISFCNANPGLMKDVAKVF
Phl_p_7_Mut    ADDMERIFKRFATAGDGKISLSELTDALRTLGSTSADEVQRMMAEIATAGDGFIDFNEFISFCNANPGLMKDVAKVF
               *********** * ******************************** * ****************************

Cyp_c_1_WT     AFAGILNDADITAALQGCQAADSFDYKSFFAKVGLSAKTPDDIKKAFAVIDQDKSGFIEEDELKLFLQNFSAGARALTDAETKAFLKAGDSDGDGKIGVDEFAALVKA
Cyp_c_1_Mut    AFAGILNDADITAALQGCQAADSFDYKSFFAKVGLSAKTPDDIKKAFAVIAQAKSGFIEEDELKLFLQNFSAGARALTDAETKAFLKAGASAGDGKIGVDEFAALVKA
               ************************************************** * ************************************ * ****************
```

Figure S2

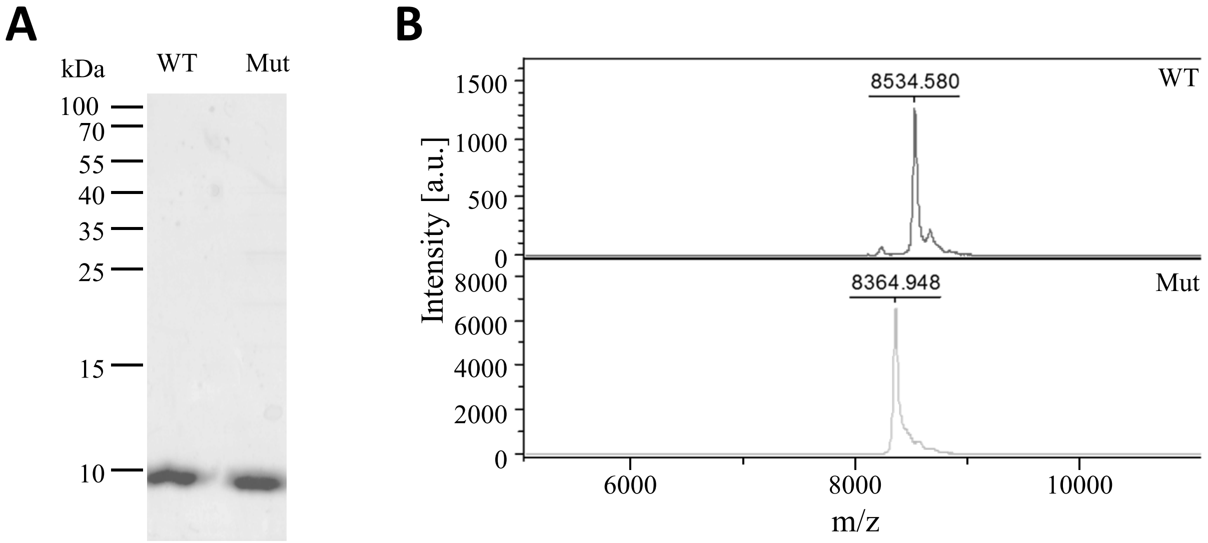
A
kDa
WT
Mut
100
70
55
40
35
25
15
10
B
Intensity [a.u.]
1500
1000
500
0
8534.580
WT
8000
6000
4000
2000
0
8364.948
Mut
6000
8000
10000
m/z

Figure S3

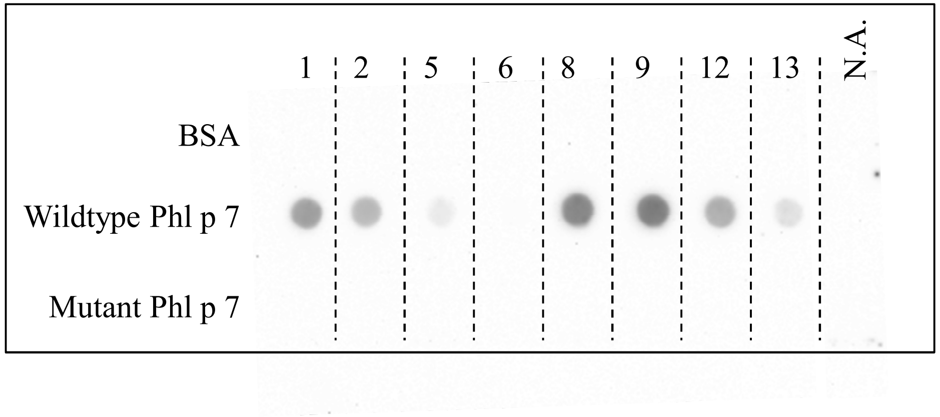

Figure S4

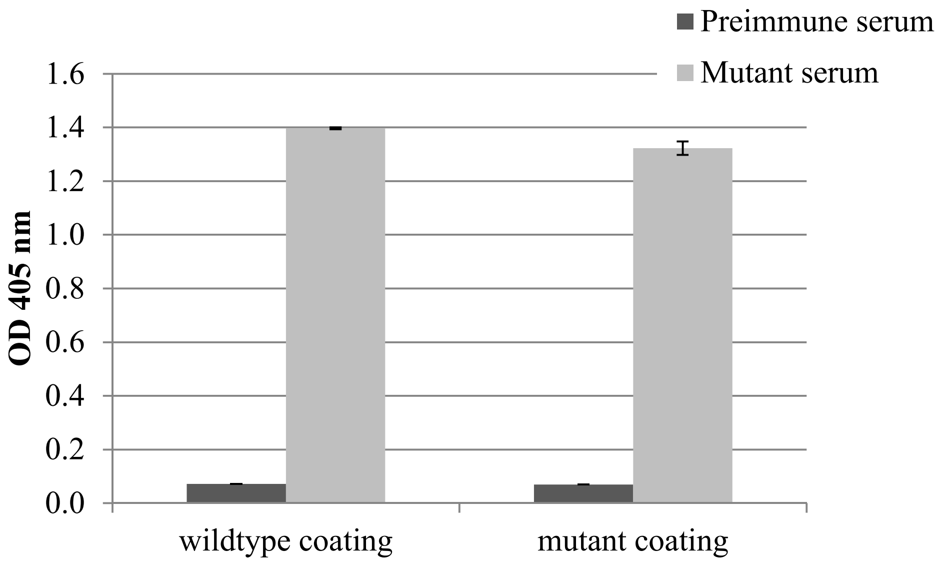
Preimmune serum
Mutant serum
OD 405 nm
1.6
1.4
1.2
1.0
0.8
0.6
0.4
0.2
0.0
wildtype coating
mutant coating
